# Identification of NLRP3^PYD^ Homo-Oligomerization Inhibitors with Anti-Inflammatory Activity

**DOI:** 10.3390/ijms23031651

**Published:** 2022-01-31

**Authors:** Soroush Moasses Ghafary, Paula M. Soriano-Teruel, Shima Lotfollahzadeh, Mónica Sancho, Eva Serrano-Candelas, Fatemeh Karami, Stephen J. Barigye, Iván Fernández-Pérez, Rafael Gozalbes, Maryam Nikkhah, Mar Orzáez, Saman Hosseinkhani

**Affiliations:** 1 Department of Nanobiotechnology, Faculty of Biological Sciences, Tarbiat Modares University, Tehran 14117-13116, Iran; s.moasses@modares.ac.ir (S.M.G.); lotfollahzade@hotmail.com (S.L.); f.karami@modares.ac.ir (F.K.); m_nikkhah@modares.ac.ir (M.N.); 2 Centro de Investigación Príncipe Felipe, Targeted Therapies on Cancer and Inflammation Laboratory, 46012 Valencia, Spain; psoriano@cipf.es (P.M.S.-T.); msancho@cipf.es (M.S.); ifernandez@cipf.es (I.F.-P.); 3 Centro de Investigación Príncipe Felipe, Polymer Therapeutics Laboratory, 46012 Valencia, Spain; 4 ProtoQSAR SL, Centro Europeo de Empresas Innovadoras, Parque Tecnológico de Valencia, 46980 Paterna, Spain; eserrano@protoqsar.com (E.S.-C.); sbarigye@moldrug.com (S.J.B.); rgozalbes@protoqsar.com (R.G.); 5 MolDrug AI Systems SL, 46018 Valencia, Spain; 6 Department of Biochemistry, Faculty of Biological Sciences, Tarbiat Modares University, Tehran 14117-13116, Iran

**Keywords:** inflammasome inhibitors, NLRP3, PYD, screening, split-luciferase, pyroptosis

## Abstract

Inflammasomes are multiprotein complexes that represent critical elements of the inflammatory response. The dysregulation of the best-characterized complex, the NLRP3 inflammasome, has been linked to the pathogenesis of diseases such as multiple sclerosis, type 2 diabetes mellitus, Alzheimer’s disease, and cancer. While there exist molecular inhibitors specific for the various components of inflammasome complexes, no currently reported inhibitors specifically target NLRP3^PYD^ homo-oligomerization. In the present study, we describe the identification of QM380 and QM381 as NLRP3^PYD^ homo-oligomerization inhibitors after screening small molecules from the MyriaScreen library using a split-luciferase complementation assay. Our results demonstrate that these NLRP3^PYD^ inhibitors interfere with ASC speck formation, inhibit pro-inflammatory cytokine IL1-β release, and decrease pyroptotic cell death. We employed spectroscopic techniques and computational docking analyses with QM380 and QM381 and the PYD domain to confirm the experimental results and predict possible mechanisms underlying the inhibition of NLRP3^PYD^ homo-interactions.

## 1. Introduction

The inflammasome represents one of the most important participants of the innate immune system [1,2]. The Nod-Like Receptor family pyrin domain containing 3 (NLRP3) inflammasome comprises the NLRP3 molecular sensor, the apoptosis-associated speck-like protein containing a caspase recruitment domain (ASC) adaptor protein (possessing PYD and CARD domains), and pro-caspase-1 [2,3,4,5,6]. The NLRP3 protein contains an N-terminal pyrin domain (NLRP3^PYD^), a central NACHT domain containing a nucleotide-binding domain (NBD) (crucial for oligomerization of NLRs upon activation), and a C-terminal leucine-rich repeat (LRR) domain [7,8]. Upon activation, the NLRP3 protein homo-oligomerizes into a disk-like architecture via NLRP3^PYD^ homo-interactions and interacts with the ASC protein via NLRP3^PYD^–ASC^PYD^ interactions. The CARD domain of ASC then recruits pro-caspase-1 CARD domain 1 to form the NLRP3-ASC-pro-caspase-1 complex/NLRP3 inflammasome [3,4,6,9,10,11]. The NLRP3 inflammasome can activate caspase-1 by responding to a wide variety of stimuli, including PAMPs (pathogen-associated molecular patterns) or DAMPs (danger-associated molecular patterns) [12]. Subsequently, caspase-1 processes two precursor proteins to form the mature pro-inflammatory cytokines, pro-interleukin 1β (pro-IL-1β) and pro-interleukin 18 (pro-IL-18), which primarily associate with innate immunity [1,13,14,15].

The broad involvement of the NLRP3 inflammasome in various inflammatory diseases makes it a highly attractive drug target. A range of pharmacological inhibitors of the NLRP3 inflammasome has been previously described, including JC124 [16], Parthenolide [17], Bay 11-7082 [18], MCC950 [19], MNS [20], CY-09 [21], Tranilast [22], OLT1177 [23], Oridonin [24], and type I Interferons (IFNs) [25].

Several drugs have been developed to target IL-1β or IL-18 to treat NLRP3-related diseases; however, the NLRP3 inflammasome activates additional pro-inflammatory events beyond IL-1β/IL-18 secretion, while other inflammasomes/inflammasome-independent pathways can promote IL-1β secretion [26,27,28].

Since NLRP3^PYD^ homo-oligomerization occurs during the initial stages of inflammasome formation, the development/identification of novel inhibitors that prevent relevant interactions (NLRP3^PYD^–NLRP3^PYD^) may have significance to the treatment of NLRP3 inflammasome-related diseases. The self-association properties of NLRP3^PYD^ and its dependence on protein and salt concentration have been studied in detail [29]. However, the contribution of NLRP3^PYD^ homo-interactions to inflammasome activation remains poorly understood. Currently described NLRP3 inhibitors such as CY-09 or MCC950 directly bind to the ATP-binding motif of the NLRP3 NACHT domain [21,30]. Although several groups have investigated various molecular inhibitors interacting with different components of inflammasome complexes [16,17,18,19,20,21,22,23,24,25,31,32,33], to the best of our knowledge, there exists no specific inhibitor of NLRP3^PYD^ homo-interactions.

In this study, we have selected candidate inhibitors of the NLRP3 inflammasome by screening a set of small molecules from the MyriaScreen Diversity Library (Sigma) with the reconstituted luciferase assay. The MyriaScreen library is produced by collaboration between TimTec, Inc. (Newark, DE, USA) and Sigma-Aldrich. This screening collection is composed of 10,000 high-purity drug-like compounds selected to maximize chemical diversity and maintain drug-like properties, which are dissolved in DMSO. 

**Figure 1:**
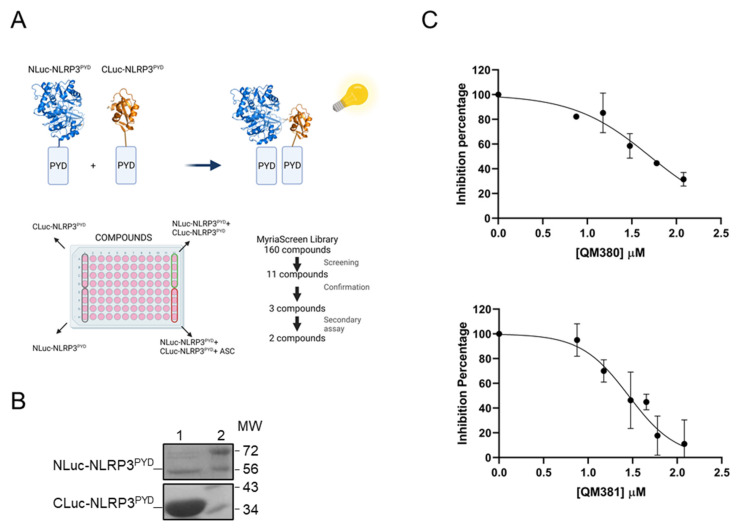
QM380 and QM381 inhibit NLRP3^PYD^ homo-oligomerization in vitro: (**A**) Flow chart describing the screening of a set of small molecules from the Myria Screen library. The first four wells of the first column of each plate were filled with CLuc-NLRP3^PYD^, and the last four wells were filled with NLuc-NLRP3^PYD^ without any compound treatment. The first four wells of the last column were used as positive controls filled with the mixture of chimeric proteins without any compound treatment, while the last four wells were considered as negative controls. (**B**) SDS-PAGE of purified N-Luc NLRP3^PYD^ and C-Luc NLRP3^PYD^ proteins. Lanes 1 in the top and bottom boxes show the purified Nluc-NLRP3^PYD^ and Cluc-NLRP3^PYD^, respectively, and Lanes 2 show the molecular weight marker (MW). (**C**) QM380 and QM381 inhibit NLRP3^PYD^ homo-oligomerization in a concentration-dependent manner. Compounds were incubated in the presence of CLuc-NLRP3^PYD^ at different concentrations for 15 min, and NLuc-NLRP3^PYD^ was then added. Luminescence was measured as described in the methods section. Luminescence data were normalized to the positive control in the absence of the compound and is expressed as the mean ± SD of *n* = 2 and *n* = 4 independent experiments for QM380 and QM381, respectively.

The split-luciferase complementary assay is based on reconstructing dissected luciferase fragments in fusion with two interacting proteins (Figure 1A). This bioluminescent assay presents the critical benefit of providing a robust signal with low background noise [34,35,36]. The optimization of both construct for drug screening is required for cell-based assays [37]. We previously utilized the split-luciferase complementary assay to detail Apaf-1–Apaf-1 interactions during apoptosome formation in vitro and in vivo [38,39,40]. Similar to the approach used in the current study, the role of different Apaf-1 domain in apoptosome formation was also revealed by the loss of function truncated and mutant forms of Apaf-1 [41,42].

This study explores QM380 and QM381 as novel modulators of the NLRP3 inflammasome that target the NLRP3^PYD^ interactions using the split-luciferase complementary assay (Figure 1A) and demonstrate their inflammasome inhibitory activity in cell-based inflammation models. We also performed spectroscopy and theoretical studies to confirm our experimental results and gain insight into the inhibition mechanisms of these two promising inflammasome inhibitors.

## 2. Results

### 2.1. Screening and Identification of Modulators of NLRP3^PYD^ Homo-Interactions Using a Split-Luciferase Assay

We expressed NLuc-NLRP3^PYD^ and CLuc-NLRP3^PYD^ and purified them to homogeneity in terms of molecular weight (as confirmed by SDS-PAGE) (Figure 1B). We selected candidate inhibitors of the NLRP3 inflammasome by screening a set of small molecules from the MyriaScreen Diversity Library (Sigma) with the reconstituted luciferase assay using purified NLuc-NLRP3^PYD^ and CLuc-NLRP3^PYD^ in a cell-free system (Figure 1A and Figure S1).

The first screen identified eleven compounds able to decrease total luciferase activity (Figure S1A). Confirmation assay selected three compounds (E3, E11, and H8) as candidate inhibitors of NLRP3 inflammasome activation (Figure S1B). We assessed the specificity of selected compounds for NLRP3^PYD^ homo-interactions in a secondary assay to eliminate direct luciferase inhibitors. The results demonstrated a decreased luminescence signal in luciferase transfected cells in the presence of H8; however, we found no significant alterations in luciferase activity in the presence of E3 or E11 (Figure S1C), confirming their specificity for PYD/PYD interactions. We confirmed the activity of E3 (QM380) and E11 (QM381) in dose-response reconstitution assays with NLuc-NLRP3^PYD^ and CLuc-NLRP3^PYD^, finding IC_50_ values of 50 and 28 μM, respectively (Figure 1C and Figure S2).

### 2.2. Structural Analysis

#### 2.2.1. Fluorescence Quenching Assay

To better understand QM381 binding to NLRP3^PYD^, we took advantage of the existence of a tryptophan residue in the sequence of this protein domain. The fluorescence intensity of the tryptophan decreased in a dose-dependent manner when we incubated the protein with increasing concentrations of QM381, indicating a change in the tryptophan environment as a consequence of compound binding produced by protein conformational rearrangement (Figure S3).

#### 2.2.2. NMR Spectroscopy

We performed WaterLOGSY and STD NMR interaction experiments to corroborate direct interactions of QM380 and QM381 with NLRP3^PYD^ (Figure S4A,B); however, we failed to achieve conclusive results in the case of QM381 due to compound solubility restrictions at the concentrations needed. Of note, both analyses demonstrated a shift in the NMR spectra, indicating the binding of QM380 to the PYD domain of NLRP3.

### 2.3. Molecular Docking Analysis

Molecular docking and structural modeling have been widely used as complementary techniques to confirm and support experimental data. To assess the appropriate binding orientations and predict the mechanism of inhibiting NLRP3^PYD^ homo-interactions and binding modes of most active compounds to NLRP3^PYD^, we performed molecular docking analysis via Autodock Vina with mentioned parameters. To the best of our knowledge, a ligand-binding site for drugs on NLRP3^PYD^ has not yet been elucidated; therefore, we assumed the interface of two monomers as a possible ligand-binding site. According to a previous study carried out by Stutz et al., the specified interfaces for homo-interaction of NLRP3^PYD^ are formed by Ser 5, Arg 7, Cys 8, Ala 11, Glu 15, Asp 50, Val 52, Asp 53, and Thr 56 residues (known as type Ιa interface) of one NLRP3^PYD^ domain and Lys 23, Lys 24, Met 27, His 28, Glu 30, and Asp 31 residues (known as type Ιb interface) of the other NLRP3^PYD^ domain [43].

**Figure 2:**
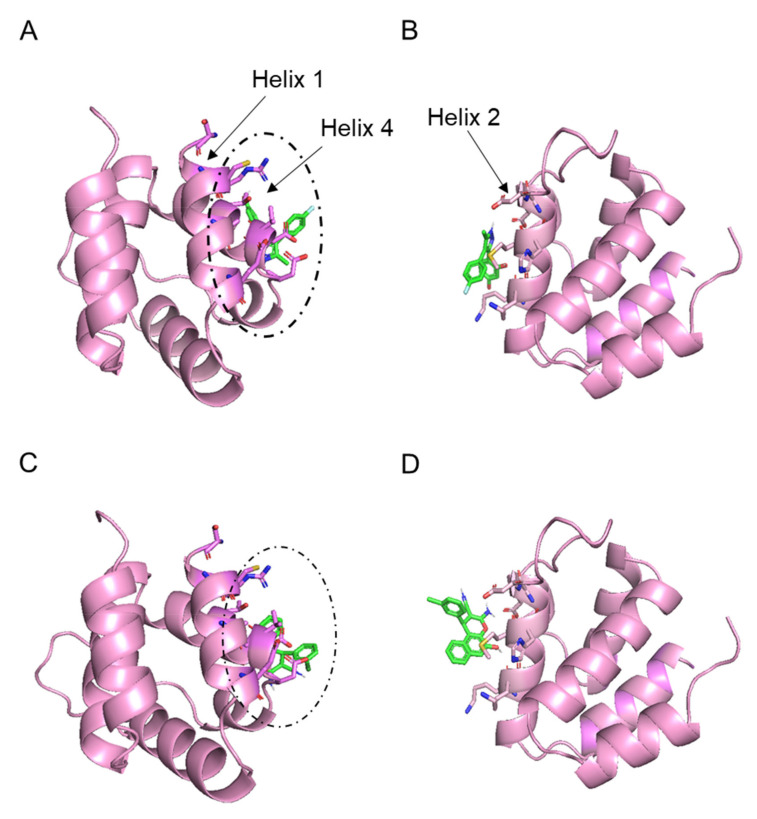
Graphical representation of docking study between NLRP3^PYD^ and (**A**,**B**) QM380, (**C**,**D**) QM381 (first poses) at the Ιa (**A**,**C**) and Ιb (**B**,**D**) interfaces, generated by Autodock Vina. In the peptide structures represented in cartoon form, residues that participated in the Ιa (helices 1 and 4) and Ιb (helix2) interfaces and ligands are in stick representation visualized by PYMOL (version.2.3.3).

Due to the type Ιa and type Ιb monomer dimerization interfaces, we carried out separate docking analyses for each site. Table 1 details the lowest binding free energy for each docking (first pose), while Figure 2A–D show the binding position of ligands to protein for the first pose for QM380 and QM381, respectively. Overall, the Ιa interface displays a slightly higher binding free energy value than the Ιb interface (Table 1). Therefore, we chose the two models with the lowest binding energy obtained by docking of each ligand against the PYD monomer, and employed the Ιa interface (Figure 2A,C) as predicted binding models for the remainder of the study.

**Figure 3:**
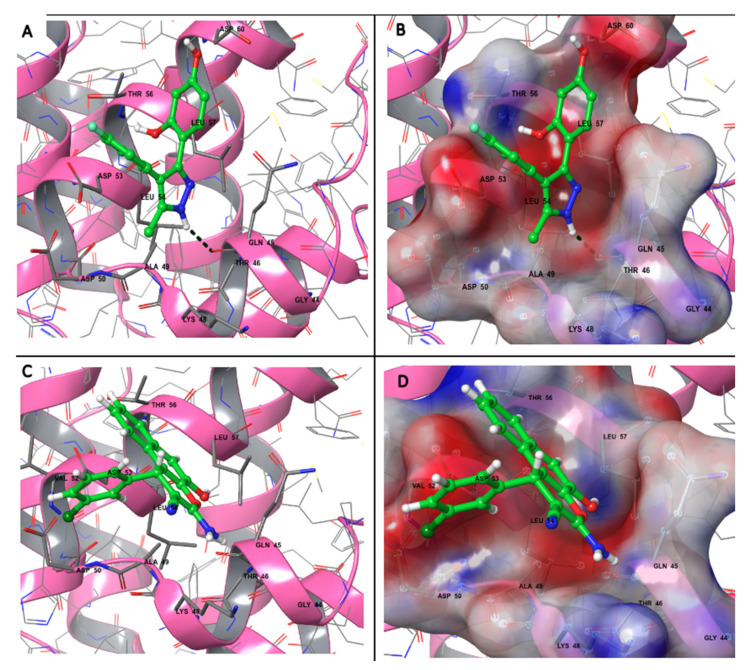
Molecular interactions and electrostatic surface potential of predicted binding models at the binding site (residues with 5 A° distance to the ligand) for (**A**,**B**) QM380 and (**C**,**D**) QM381. The residues that interact are labeled, visualized in the ball–stick model, and colored based on their physicochemical properties. The hydrogen bond of Gln 45 and QM380 is shown as a dashed line. In panels (**B**,**D**), the surface ranges are characterized from positive electrostatic potential (blue surface) to a negative potential (red surface) with 30% transparency. The structures and interfaces were represented and analyzed by Maestro (version 12.6).

To gain further insight into the predicted binding models by Autodock Vina, we performed a binding orientation analysis of the binding site (residues with 5 Å distance to the ligand) using Maestro (Figure 3). Figure 3 illustrates the participating residues in binding sites for QM380 and QM381; both charged residues (e.g., Lys and Asp) or polar residues (e.g., Gln and Thr) surround both ligands possibly due to the electronegative atoms (fluorine and chlorine) or amine, ether, and hydroxyl functional groups of the compounds. Furthermore, the program predicted one hydrogen bond involving the oxygen atom of Gln 45 and the hydrogen atom (connected to the nitrogen atom) of QM380; however, the presence of hydrophobic residues in the binding sites (e.g., Ala, Val, and Leu) indicates the involvement of both electrostatic and hydrophobic interactions in ligand binding to NLRP3^PYD^.

### 2.4. NLRP3^PYD^ Homo-Oligomerization Inhibitors Prevent ASC Speck Formation

After confirming the ability of QM380 and QM381 to inhibit NLRP3^PYD^ homo-oligomerization in a cell-free in vitro experimental system, we next aimed to explore the ability of these compounds to inhibit the formation of ASC specks and mature caspase-1 and, thus, interfere with inflammasome function in the cellular milieu. We studied the ability of QM380 and QM381 to inhibit the formation of ASC specks in THP1-ASC-GFP cells, a cell line derived from THP-1 human monocytic cells that stably express an ASC-GFP fusion protein under the control of the NF-kB promoter. LPS treatment activates the fusion protein expression in these cells, which can be visualized throughout the cell cytoplasm; a subsequent second signal (NLRP3 inflammasome activation by nigericin treatment) then causes ASC-GFP speck formation, whose relative levels can be determined by confocal fluorescence microscopy.

**Figure 4:**
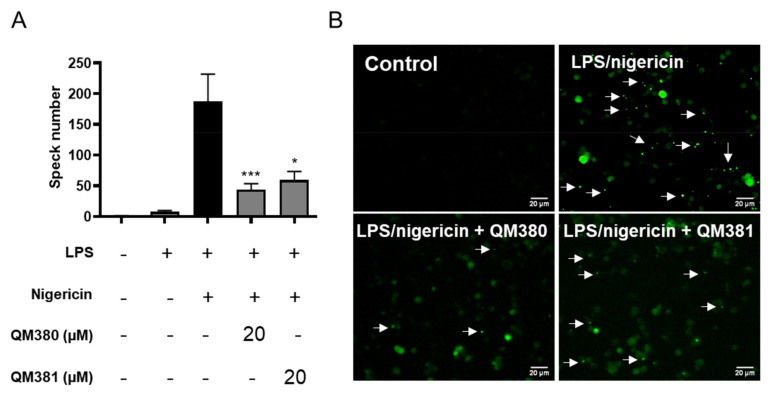
QM380 and QM381 inhibit ASC speck formation: (**A**) Percentage of ASC specks measured in THP-1-ASC-GFP cells treated with QM380 and QM381 (20 µM) and stimulated with LPS (100 ng/mL) and nigericin (10 µM). (**B**) Live-cell imaging of THP-1-ASC-GFP cells treated as indicated above. Scale bar corresponds to 20 µm. Arrows point to ASC specks. Asterisks represent significant differences compared to the stimulated control (LPS/nigericin) as determined by a one-way ANOVA test with Tukey’s multiple post-test comparisons * *p* < 0.05; *** *p* < 0.001. All data are expressed as the mean ± SD of four independent experiments.

We monitored ASC-GFP oligomerization following LPS and nigericin treatment in the presence or absence of the NLRP3^PYD^ homo-oligomerization inhibitors. Interestingly, both QM380- and QM381-treated samples displayed a significant reduction in ASC speck formation after the appropriate stimulation (Figure 4). These results support the contribution of NLRP3^PYD^ as a nucleation site for inflammasome activation and reinforce the use of this domain as a target for drug development.

### 2.5. Inhibition of NLRP3^PYD^ Homo-Oligomerization with QM380 and QM381

**Figure 5:**
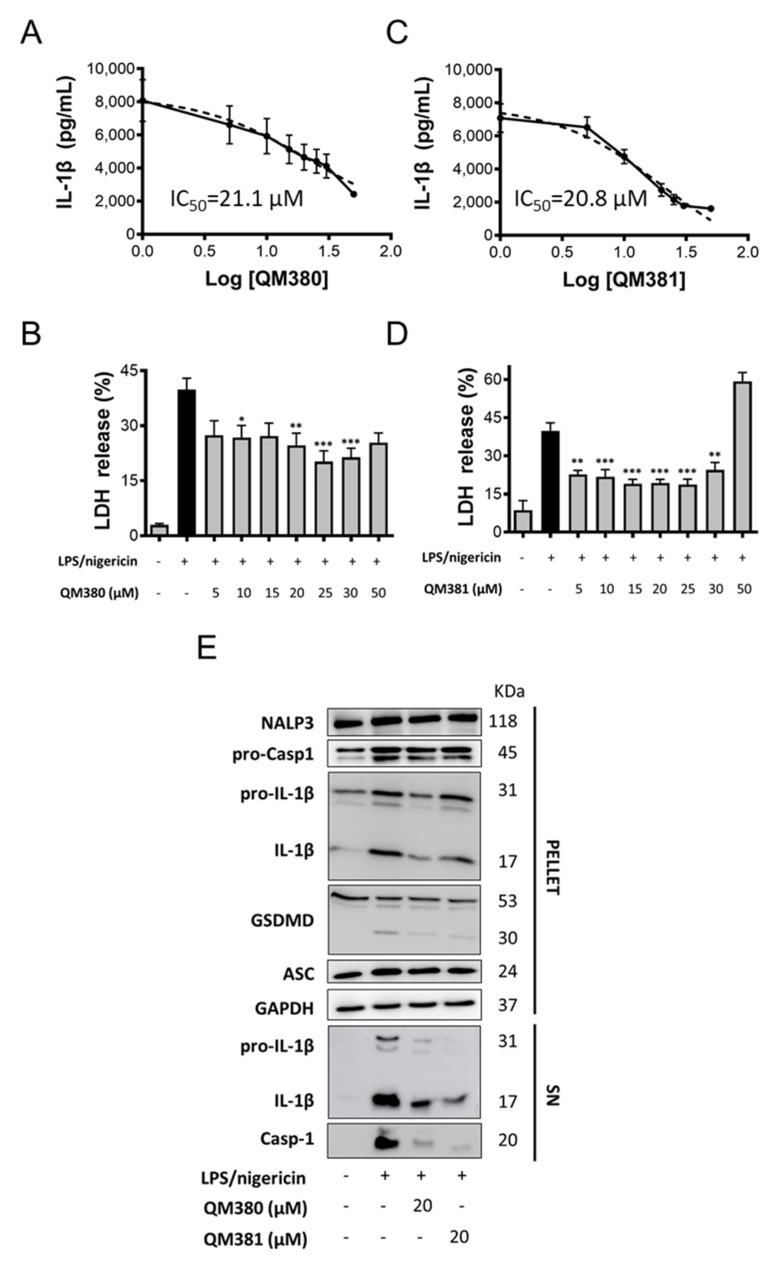
QM380 and QM381 inhibit NLRP3 activation mediated by LPS and nigericin stimulation in PMA-differentiated THP-1 cells. The ELISA technique evaluated IL-1β secretion upon activation of the NLRP3 inflammasome with LPS (100 ng/mL) and nigericin (20 µM) and treated or not with QM380 (**A**) or QM381 (**B**) at different concentrations. Measurement of LDH release under the above-described conditions for QM380 (**C**) and QM381 (**D**) treated cells. (**E**) THP-1 cells were stimulated as described above, and supernatants (SN) and pellets were analyzed by immunoblotting for IL-1β and cleaved caspase-1. A representative Western blot is shown. Asterisks represent significant differences compared to the stimulated control (LPS/nigericin) as determined by a one-way ANOVA test with Tukey’s multiple post-test comparisons * *p* < 0.05; ** *p* < 0.01; *** *p* < 0.001. All data are expressed as the mean ± SD of three experiments.

Reduction in ASC oligomerization and subsequent inhibition of NLRP3 inflammasome activity were evaluated by the QM380 and QM381 effect on human macrophages pre-treated with LPS and nigericin to induce NLRP3 inflammasome activation. We monitored inflammasome activation via the secretion of the pro-inflammatory cytokine IL-1β and pyroptotic cell death (Figure 5). Encouragingly, both QM380 and QM381 treatment inhibited NLRP3 activation, as evidenced by a decrease in IL-1β secretion (Figure 5A,B, respectively) and reduced levels of pyroptosis, as measured by a decreased release of lactate dehydrogenase (LDH) (Figure 5C,D, respectively). Cell death data (Figure S5) and GSDMD cleavage by immunoblotting (Figure 5E) confirm recovery from pyroptotic death.

Analysis of the zymogen (pro-) and processed mature forms of caspase-1 and IL-1β in cellular extracts (Figure 5E—PELLET fraction) and supernatants (Figure 5E—SN fraction) by immunoblotting demonstrated a reduction in caspase-1 and IL-1β processing in the presence of QM380 and QM381, which agreed with the IL-1 β secretion assays. Overall, this suggests that NLRP3^PYD^ oligomerization inhibitors can decrease pro-inflammatory signaling and pro-inflammatory cell death, thereby inhibiting the spread of inflammation.

## 3. Discussion

Involvement of the NLRP3 inflammasome in a wide range of pathological processes [44,45,46,47,48] requires a deeper understanding of the protein–protein interaction processes that modulate its activation for therapeutic intervention.

Different inhibitory mechanisms have been investigated using diverse inhibitors that counteract NLRP3 inflammasome assembly [49]. Indirect inhibitors of NLRP3 inflammasome activation include Glyburide, Bay 11-7082, β-hydroxybutyrate, VX-740, and JC124, which function by inhibiting ATP-sensitive K^+^ channels, inhibiting the NF-κB pathway by inhibiting the kinase activity of IKKβ, K^+^ efflux, blocking caspase-1 and reducing the expression of NLRP3, ASC, and caspase-1, respectively [50,51,52,53,54], and indirect inhibitors such as Parthenolide and Bay 11-7082 [18,55].

Moreover, direct inhibitors for NATCH domain (e.g., INF39, 3, 4-methylenedioxy-b-nitrostyrene, CY-09, Dapansutrile, MCC950, and Oridonin) and β-carotene are reported [20,21,24,56,57,58,59].

Here, we demonstrated that QM380 and QM381 compounds directly interfered with NLRP3^PYD^ homo-oligomerization to decrease ASC oligomer formation and inflammatory signaling activation (Figure 4). These results provide evidence for the NLRP3^PYD^ domain as a homo-oligomerization seed platform for the formation of ASC specks.

Many recent studies focusing on inhibiting inflammasomal complexes by small molecules have provided mechanistic insight using in silico studies [21,32,59,60,61,62,63,64]. Thus, to illuminate the mechanisms of QM380 and QM381, we performed spectroscopic and molecular docking studies. As there is no revealed specified binding site for NLRP3^PYD^, we have considered the residues involved in the interface of two monomers as a possible ligand-binding site. Results of this analysis and our experimental findings reveal that both inhibitors most likely bind to the interface of interaction of the two monomers and, by interacting with some of the residues at this interface, disrupt interaction between the two NLRP3^PYD^s [43]. Fluorescence and NMR spectroscopies (Figures S3 and S4) demonstrated conformational changes in the PYD domain or oligomerization pattern changes, presumably induced through direct ligand binding. Docking results further supported the experimental data and predicted that QM380 and QM381 most likely bind to the interface controlling the NLRP3^PYD^ homo-interaction (most probably the Ιa interface with stronger affinity) mostly through electrostatic interactions, which consequently disrupt the interaction of NLRP3^PYD^ monomers. The structural and docking analyses suggest that, through direct interaction or disruption of NLRP3^PYD^ homo-oligomerization, QM380 and QM381 disrupt the correct positioning of NLRP3^PYD^ thereby decreasing inflammasome complex formation, as confirmed by analysis of speck formation (Figure 4), caspase-1 activity, GSDMD cleavage, and pyroptotic cell death, which is confirmed by the inhibition of LDH release from cells. Our study suggests that QM380 and QM381 represent promising hit compounds that may support the development of novel, more efficient inflammasome inhibitors via virtual screening studies.

## 4. Materials and Methods

*E*. *coli* BL21(DE3) was obtained from MerckMillipore (Burlington, MA, USA). pET28a expression vector was purchased from Invitrogen (Waltham, MA, USA). Fetal bovine serum (FBS) and RPMI 1640 were purchased from Gibco, Thermo Fisher Scientific (Waltham, MA, USA). Plasmid extraction kit and Ni-NTA-agarose column were obtained from Qiagen (Hilden, Germany). The luciferase assay kit was purchased from Promega (Madison, WI, USA).

### 4.1. Cell-Free Assays

#### 4.1.1. Preparation of Constructs

As described in our previous study in detail [36], N and C-terminal domains of firefly luciferase were fused to NLRP3^PYD^ to produce NLuc-NLRP3^PYD^ and CLuc-NLRP3^PYD^ recombinant proteins, respectively. A bioluminescence signal was observed in the case of NLRP3^PYD^ homo-interactions and the reconstitution of the complete luciferase protein.

#### 4.1.2. Expression and Purification of Chimeric Proteins

The desired proteins were expressed in *E*. *coli* and purified, as previously described [36]. Briefly, the constructs cloned in pET28a were transformed into *E*. *coli* BL21(DE3). Then, 10 mL of LB medium containing 50 mM kanamycin was incubated with a fresh bacterial colony harboring the expression plasmid, and grown at 37 °C overnight under shaking at 180 rpm. Then, 1 mL of pre-cultured bacteria was used to inoculate 250 mL 2xyt medium and grown at 37 °C under shaking until the appropriate OD_600_ (0.6) was reached. The mixture was induced by isopropyl-β-D-1-thiogalactopyranoside (IPTG) and incubated at 37 °C under shaking. The cells were harvested by centrifugation at 6000 rpm for 20 min at 4 °C. The cell pellet was resuspended in lysis buffer (20 mM Tris-Base, 500 mM NaCl, 8 M urea, 5 mM imidazole) and sonicated on ice in ten cycles of 10 s bursts and 20 s rest intervals. The lysate was centrifuged at 12,000 rpm for 20 min. Then the supernatant was loaded onto a Ni-NTA-agarose column and incubated. The column was washed using several buffers (20 mM Tris/HCl, 500 mM NaCl, 5 mM Imidazole). Finally, the desired proteins were eluted from the column by elution buffer (20 mM Tris-HCl, 300 mM NaCl, and 275 mM imidazole; pH 7.8). Following electrophoresis, the gels were incubated in a stain solution containing 40% (*v*/*v*) methanol, 10% (*v*/*v*) glacial acetic acid, and 0.1% (*w*/*v*) Coomassie Brilliant Blue R-250 in distilled water at room temperature for 1 h under gentle agitation. Then, the Coomassie stain was removed by destain solution containing 40% (*v*/*v*) methanol and 10% (*v*/*v*) acetic acid in distilled water. Representative image was obtained after gel scanning in 8-bit grayscale. Protein size corresponds to the predicted molecular weight (the molecular weight of Cluc-NLRP3^PYD^ and Nluc-NLRP3^PYD^ are approximately 33 KDa and 60 kDa, respectively). Then, proteins were dialyzed against PBS buffer at pH 7.4 and stored at −80 °C for subsequent steps.

#### 4.1.3. Screening of Small Molecule Compounds

To target specific NLRP3^PYD^ homo-interactions by small compound molecules, random screening of 160 small molecules from the MyriaScreen Diversity Library (Sigma) was carried out using a firefly luciferase assay. The best candidates were selected as compounds for further assessment in vitro. The assay was carried out in white 96-well plates. First, CLuc-NLRP3^PYD^ (~16 µg) was added to each well containing 20 mM Tris buffer, then each library compound and, finally, NLuc-NLRP3^PYD^ (~8 µg) were loaded. The first four wells of the first column of each plate were filled with CLuc-NLRP3^PYD^, and the last four wells were filled with NLuc-NLRP3^PYD^ without any compound treatment. The first four wells of the last column were used as positive controls filled with the mixture of chimeric proteins without any compound treatment, while the last four wells were considered as negative controls and filled with the mixture of CLuc-NLRP3^PYD^, purified human ASC protein (~4 µg), and NLuc-NLRP3^PYD^ (flow chart is shown in Figure 1A). The equivalent volume of DMSO was added in each positive and negative well. Next, 50 µL of luciferase assay reagent from the Promega kit was added to each well, and the luminescence signal was recorded in a CLARIOstar Plus Multi-Mode Microplate Reader. The quality of the screening was assessed by calculating the z-factor according to the following equation:

z = 1 − [3 × (standard deviation of positive controls) + 3 × (standard deviation of negative controls)] / [(average of positive controls) − (average of negative controls)]

The first screen identified eleven compounds able to decrease total luciferase activity (Figure S1A). Confirmation assay was carried out to select candidate compounds as inhibitors of NLRP3 inflammasome activation (Figure S1B).

### 4.2. Structural Analysis

#### 4.2.1. Fluorescence Quenching Assay

The tryptophan fluorescence quenching assay can be used to assess small molecule inhibitor binding affinities to proteins using fluorescence spectroscopy [65].

Tryptophan fluorescence quenching assays were performed in black 96-well plates. The CLuc-NLRP3^PYD^ chimeric protein was added to each well, and then different concentrations (2, 10, 20, and 50 µM) of QM381 compound were loaded in the wells. The fluorescent signal of each well was detected at the same time. NLRP3^PYD^ excitation at 280 nm prompts an emission maximum corresponding to the tryptophan fluorescence at 352 nm in a Jasco FP-8500 spectrofluorometer.

#### 4.2.2. NMR Spectroscopy

To confirm the interaction between proteins and small molecule inhibitors, WaterLOGSY and STD NMR interaction experiments with the compounds were performed in the NMR facility of the CIPF.

#### 4.2.3. Structure Refinement, Active Site Analysis, and Docking Simulation Study

The structure of QM380 and QM381 were downloaded from the ZINC database in mol2 format [66], and the crystal structure of the NLRP3^PYD^ peptide (PDB ID: 3QF2) was downloaded from the Research Collaboratory for Structural Bioinformatics (RCSB) Protein Data Bank (PDB) [67]. All structures were refined and modified by removing the solvent, adding polar hydrogen for proteins, merging non-polar hydrogens with the corresponding carbons, defining rotatable bonds for ligands, and assigning Gasteiger charges before being converted into the pdbqt format using Autodock tools (version 1.5.6) [68].

An in silico docking simulation study was carried out to evaluate the potential of chemical compounds to inhibit NLRP3^PYD^ homo-interactions. The interface of two monomers (PDB ID: 3QF2 chainA) was predicted as the ligand-binding site. Due to the two different interfaces for each monomer (Ιa and Ιb interfaces) [43], two grid boxes (box center x: −24.281, y: 19.557, z: 28.099) for type Ιa interaction and (box center x: −12.659, y: 34.025, z: 29.694) for type Ιb interaction were designed using Autodock tools. The grid box size was set to 25 × 25 × 25 A° and was kept constant for all site-specific dockings. All dockings were performed by Autodock Vina (version 1.1.2) [69]. The output results were in PDBQT format and converted to PDB using the UCSF Chimera software (Version.1.15) [70]. The most favorable docking pose for each ligand was chosen for the subsequent analysis.

All structures and docking results were visualized using PYMOL (version.2.3.3) [71] and Maestro (version 12.6, Schrodinger, LLC, New York, NY, USA, 2020).

Poisson–Boltzmann electrostatic potential surfaces, peptide-ligand binding site, and molecular interactions at the interface were analyzed and visualized using Maestro.

### 4.3. Cell-Based Assays

#### 4.3.1. Evaluation of the Specificity of Selected Inhibitors to NLRP3^PYD^ Homo-Interactions

To determine the specificity of chemical compounds for NLRP3^PYD^ homo-interactions and not to NLuc or CLuc fragments, the luciferase transfected cell lysate was exposed to the selected small molecule compounds and the luminescent signal measured. For this reason, luciferase transfected HEK293 cells at a density of 5 × 10^5^ cells in each well of a six-well plate were gently washed twice with PBS, and the cells were then lysed with 50 µL Cell Culture Lysis Reagent (CCLR) buffer. The lysate of luciferase transfected cells was added to each well of a white plate, and the selected compounds (E3, E11, and H8) from screening (Figure S1C) were added separately. The luminescent signal of each well was recorded (RLU/sec) in the presence of firefly luciferase substrate in a CLARIOstar Plus Multi-Mode Microplate Reader.

#### 4.3.2. ASC Speck Assay

THP1-ASC-GFP cells were seeded at 1 × 10^6^/mL and differentiated into macrophages with 5 ng/mL of phorbol-12-myristate-13-acetate (PMA) the day prior to use in experiments on 35 mm glass-bottom culture dishes. The following day, the medium was replaced with a medium containing inhibitors (20 µM) or vehicle (DMSO) for 30 min. Cells were then primed with 100 ng/mL lipopolysaccharide (LPS) for 3 h and stimulated with 10 µM nigericin for 30 min. Images were acquired using a Leica DM 6000 microscope (Leica DC500 camera) with a 10× objective. The number of ASC specks was registered for a total of 40 images per condition (ten random fields for each condition in four independent experiments). For confocal analysis (Leica SP8), samples were fixed in 4% paraformaldehyde (PFA) for 10 min at room temperature, washed several times, and prepared in mounting medium plus DAPI. Image processing was performed using FiJi software.

#### 4.3.3. Inflammasome Activation Assay

Inhibitors were evaluated in the PMA-differentiated THP-1 cells stimulated with 100 ng/mL LPS and 20 µM nigericin to stimulate the NLRP3 inflammasome. Briefly, 1 × 10^6^ cells were seeded in 6-well plates in 1 mL RPMI media (1% FBS). Cells were either mock-treated or primed with compounds at the indicated concentrations for 30 min, followed by treatment with LPS for 3 h and then nigericin for 30 min at 37 °C. Supernatants were harvested and clarified by centrifugation at 1500 rpm at room temperature, and cytokine analysis was performed. IL-1β secretion was monitored by ELISA assay (BD OptEIA™ Human IL-1β ELISA Kit) following the manufacturer’s instructions. Cell viability was analyzed in parallel by evaluating the release of lactate dehydrogenase (LDH) according to a commercial kit (CytoTox-ONE™ Homogeneous Membrane Integrity Assay; Promega). The release of LDH was calculated using the formula: the release of LDH (%) = 100 × (Abs490 treated − Abs490 untreated cells)/Abs490 untreated cells lysed with Triton 9% (maximum release of LDH).

### 4.4. Immunoblotting

The supernatants of the treated cells were precipitated using the chloroform–methanol method, as described by De Nardo. et al. [72]. Pellets were obtained by lysing cells in 25 mM of Tris-HCl pH 7.4, 1 mM EDTA, 1 mM EGTA, and 1% SDS, plus protease and phosphatase inhibitors. BCA protein assay was used to determine protein concentration. Samples were separated in a 14% SDS-PAGE gel, transferred to a nitrocellulose membrane, and blocked with 5% skimmed milk for 1 h. Then, the membrane was incubated overnight with primary antibodies: α-casp1 (1:1000; Cell Signaling 2225), α-IL-1β (1:1000; Cell Signaling #82186S and Merck Millipore #MAB18), α-NLRP3 (1:1000; Cell Signaling 15101), α-ASC (1:1000; sc-22514), α-GSDMD (1:1000; Cell Signaling #93709), and α-GAPDH (1:1000; Cell Signaling 2118S) at 4 °C. Membranes were washed and probed with the appropriate secondary antibody conjugated with peroxidase for enhanced chemiluminescence detection (GE Healthcare Bio Sciences AB, Uppsala, Sweden).

## Figures and Tables

**Table 1:** The lowest binding free energy (kcal/mol) obtained from docking of two different interfaces of NLRP3^PYD^ with the most active ligands using the Autodock Vina tool.

Compounds	Interface Type	The Lowest Binding Free Energy (kcal/mol)
QM380	Ιa interface	−5.8
	Ιb interface	−4.9
QM381	Ιa interface	−5.9
	Ιb interface	−4.9

## Data Availability

Data available on request from the authors.

## Supplementary Materials

The following are available online at https://www.mdpi.com/article/10.3390/ijms23031651/s1.

## References

[1] Martinon F., Burns K., Tschopp J. (2002). The inflammasome: A molecular platform triggering activation of inflammatory caspases and processing of proIL-β. Mol. Cell..

[2] Schroder K., Zhou R., Tschopp J. (2010). The NLRP3 inflammasome: A sensor for metabolic danger?. Science.

[3] Jin C., Flavell R.A. (2010). Molecular mechanism of NLRP3 inflammasome activation. J. Clin. Immunol..

[4] Sutterwala F.S., Haasken S., Cassel S.L. (2014). Mechanism of NLRP3 inflammasome activation. Ann. N. Y. Acad. Sci..

[5] Swanson K.V., Deng M., Ting J.P.-Y. (2019). The NLRP3 inflammasome: Molecular activation and regulation to therapeutics. Nat. Rev. Immunol..

[6] Jo E.K., Kim J.K., Shin D.M., Sasakawa C. (2016). Molecular mechanisms regulating NLRP3 inflammasome activation. Cell. Mol. Immunol..

[7] Jha S., Ting J.P.Y. (2009). Inflammasome-associated nucleotide-binding domain, leucine-rich repeat proteins and inflammatory diseases. J. Immunol..

[8] Bertin J., DiStefano P. (2000). The PYRIN domain: A novel motif found in apoptosis and inflammation proteins. Cell Death Differ..

[9] Srinivasula S.M., Poyet J.L., Razmara M., Datta P., Zhang Z., Alnemri E.S. (2002). The PYRIN-CARD protein ASC is an activating adaptor for caspase-1. JBC.

[10] Zheng D., Liwinski T., Elinav E. (2020). Inflammasome activation and regulation: Toward a better understanding of complex mechanisms. Cell Discov..

[11] Broz P., Dixit V.M. (2016). Inflammasomes: Mechanism of assembly, regulation and signalling. Nat. Rev. Immunol..

[12] Winsor N., Krustev C., Bruce J., Philpott D.J., Girardin S.E. (2019). Canonical and noncanonical inflame masomes in intestinal epithelial cells. Cell Microbiol..

[13] Kopitar-Jerala N. (2017). The role of interferons in inflammation and inflammasome activation. Front. Immunol..

[14] Licandro G., Ling Khor H., Beretta O., Lai J., Derks H., Laudisi F., Conforti-Andreoni C., Liang Qian H., Gee Teng G., Ricciardi-Castagnoli P. (2013). The NLRP 3 inflammasome affects DNA damage responses after oxidative and genotoxic stress in dendritic cells. Eur. J. Immunol..

[15] Bergsbaken T., Fink S.L., Cookson B.T. (2009). Pyroptosis: Host cell death and inflammation. Nat. Rev. Microbiol..

[16] Marchetti C., Toldo S., Chojnacki J., Mezzaroma E., Liu K., Salloum F.N., Nordio A., Carbone S., Mauro A.G., Das A. (2015). Pharmacologic inhibition of the NLRP3 inflammasome preserves cardiac function after ischemic and non-ischemic injury in the mouse. J. Cardiovasc. Pharmacol..

[17] Heinrich M., Robles M., West J.E., Ortiz de Montellano B.R., Rodriguez E. (1998). Ethnopharmacology of Mexican asteraceae (compositae). Ann. Rev. Pharmacol. Toxicol..

[18] Juliana C., Fernandes-Alnemri T., Wu J., Datta P., Solorzano L., Yu J.W., Meng R., Quong A.A., Latz E., Scott C.P. (2010). Anti-inflammatory compounds parthenolide and Bay 11-7082 are direct inhibitors of the inflammasome. JBC.

[19] Jiao J., Zhao G., Wang Y., Ren P., Wu M. (2020). MCC950, a selective inhibitor of NLRP3 inflammasome, reduces the inflammatory response and improves neurological outcomes in mice model of spinal cord injury. Front. Mol. Biosci..

[20] He Y., Varadarajan S., Muñoz-Planillo R., Burberry A., Nakamura Y., Núñez G. (2014). 3, 4-methylenedioxy-β-nitrostyrene inhibits NLRP3 inflammasome activation by blocking assembly of the inflammasome. JBC.

[21] Jiang H., He H., Chen Y., Huang W., Cheng J., Ye J., Wang A., Tao J., Wang C., Liu Q. (2017). Identification of a selective and direct NLRP3 inhibitor to treat inflammatory disorders. J. Exp. Med..

[22] Huang Y., Jiang H., Chen Y., Wang X., Yang Y., Tao J., Deng X., Liang G., Zhang H., Jiang W. (2018). Tranilast directly targets NLRP 3 to treat inflammasome-driven diseases. EMBO Mol. Med..

[23] Lonnemann N., Hosseini S., Marchetti C., Skouras D.B., Stefanoni D., D’Alessandro A., Dinarello C.A., Korte M. (2020). The NLRP3 inflammasome inhibitor OLT1177 rescues cognitive impairment in a mouse model of Alzheimer’s disease. Proc. Natl. Acad. Sci. USA.

[24] He H., Jiang H., Chen Y., Ye J., Wang A., Wang C., Liu Q., Liang G., Deng X., Jiang W. (2018). Oridonin is a covalent NLRP3 inhibitor with strong anti-inflammasome activity. Nat. Commun..

[25] Guarda G., Braun M., Staehli F., Tardivel A., Mattmann C., Förster I., Farlik M., Decker T., Du Pasquier R.A., Romero P. (2011). Type I interferon inhibits interleukin-1 production and inflammasome activation. Immunity.

[26] Lu B., Nakamura T., Inouye K., Li J., Tang Y., Lundbäck P., Valdes-Ferrer S.I., Olofsson P.S., Kalb T., Roth J. (2012). Novel role of PKR in inflammasome activation and HMGB1 release. Nature.

[27] Yang Y., Wang H., Kouadir M., Song H., Shi F. (2019). Recent advances in the mechanisms of NLRP3 inflammasome activation and its inhibitors. Cell Death Dis..

[28] Nowarski R., Jackson R., Gagliani N., de Zoete M.R., Palm N.W., Bailis W., Low J.S., Harman C.C., Graham M., Elinav E. (2015). Epithelial IL-18 equilibrium controls barrier function in colitis. Cell.

[29] Oroz J., Barrera-Vilarmau S., Alfonso C., Rivas G., de Alba E. (2016). ASC pyrin domain self-associates and binds NLRP3 protein using equivalent binding interfaces. JBC.

[30] Coll R.C., Hill J.R., Day C.J., Zamoshnikova A., Boucher D., Massey N.L., Chitty J.L., Fraser J.A., Jennings M.P., Robertson A.A. (2019). MCC950 directly targets the NLRP3 ATP-hydrolysis motif for inflammasome inhibition. Nat. Chem. Biol..

[31] Sborgi L., Ude J., Dick M.S., Vesin J., Chambon M., Turcatti G., Broz P., Hiller S. (2018). Assay for high-throughput screening of inhibitors of the ASC-PYD inflammasome core filament. Cell Stress.

[32] Pal A., Neo K., Rajamani L., Ferrer F.J., Lane D.P., Verma C.S., Mortellaro A. (2019). Inhibition of NLRP3 inflammasome activation by cell-permeable stapled peptides. Sci. Rep..

[33] Sušjan P., Lainšček D., Strmšek Ž., Hodnik V., Anderluh G., Hafner-Bratkovič I. (2020). Selective inhibition of NLRP3 inflammasome by designed peptide originating from ASC. FASEB J..

[34] Azad T., Tashakor A., Hosseinkhani S. (2014). Split-luciferase complementary assay: Applications, recent developments, and future perspectives. Anal. Bioanal. Chem..

[35] Torkzadeh-Mahani M., Ataei F., Nikkhah M., Hosseinkhani S. (2012). Design and development of a whole-cell luminescent biosensor for detection of early-stage of apoptosis. Biosens. Bioelectron..

[36] Isazadeh M., Amandadi M., Haghdoust F., Lotfollazadeh S., Orzáez M., Hosseinkhani S. (2022). Split-luciferase complementary assay of NLRP3 PYD-PYD interaction indicates inflammasome formation during inflammation. Anal. Biochem..

[37] Oladzad A., Nikkhah M., Hosseinkhani S. (2020). Optimization of experimental variables influencing apoptosome biosensor in HEK293T cells. Sensors.

[38] Noori A.R., Hosseini E.S., Nikkhah M., Hosseinkhani S. (2018). Apoptosome formation upon overexpression of native and truncated Apaf-1 in cell-free and cell-based systems. Arch. Biochem. Biophys..

[39] Hosseini E.S., Nikkhah M., Hamidieh A.A., Fearnhead H.O., Concordet J.P., Hosseinkhani S. (2020). The Lumiptosome, an engineered luminescent form of the apoptosome can report cell death by using the same Apaf-1 dependent pathway. J. Cell Sci..

[40] Tashakor A., H-Dehkordi M., O’Connell E., Gomez Ganau S., Gozalbes R., Eriksson L.A., Hosseinkhani S., Fearnhead H.O. (2019). A new split-luciferase complementation assay identifies pentachlorophenol as an inhibitor of apoptosome formation. FEBS Open Bio.

[41] Noori A.-R., Tashakor A., Nikkhah M., Eriksson L.A., Hosseinkhani S., Fearnhead H.O. (2021). Loss of WD2 subdomain of Apaf-1 forms an apoptosome structure which blocks activation of caspase-3 and caspase-9. Biochimie.

[42] Sahebazzamani F., Hosseinkhani S., Eriksson L.A., Fearnhead H.O. (2021). Apoptosome formation through disruption of the K192-D616 salt bridge in the Apaf-1 closed form. ACS Omega.

[43] Stutz A., Kolbe C.C., Stahl R., Horvath G.L., Franklin B.S., van Ray O., Brinkschulte R., Geyer M., Meissner F., Latz E. (2017). NLRP3 inflammasome assembly is regulated by phosphorylation of the pyrin domain. J. Exp. Med..

[44] Latz E., Xiao T.S., Stutz A. (2013). Activation and regulation of the inflammasomes. Nat. Rev. Immunol..

[45] Song L., Pei L., Yao S., Wu Y., Shang Y. (2017). NLRP3 inflammasome in neurological diseases, from functions to therapies. Front. Cell. Neurosci..

[46] Liu-Bryan R., Terkeltaub R. (2011). Tophus biology and pathogenesis of monosodium urate crystal–induced inflammation. Gout Other Cryst. Arthropathies.

[47] Abderrazak A., Syrovets T., Couchie D., El Hadri K., Friguet B., Simmet T., Rouis M. (2015). NLRP3 inflammasome: From a danger signal sensor to a regulatory node of oxidative stress and inflammatory diseases. Redox Biol..

[48] Fusco R., Siracusa R., Genovese T., Cuzzocrea S., Di Paola R. (2020). Focus on the role of NLRP3 inflammasome in diseases. Int. J. Mol. Sci..

[49] Zahid A., Li B., Kombe A.J., Jin T., Tao J. (2019). Pharmacological inhibitors of the NLRP3 inflammasome. Front. Immunol..

[50] Lamkanfi M., Mueller J.L., Vitari A.C., Misaghi S., Fedorova A., Deshayes K., Lee W.P., Hoffman H.M., Dixit V.M. (2009). Glyburide inhibits the Cryopyrin/Nalp3 inflammasome. J. Cell Biol..

[51] Strickson S., Campbell D.G., Emmerich C.H., Knebel A., Plater L., Ritorto M.S., Shpiro N., Cohen P. (2013). The anti-inflammatory drug BAY 11-7082 suppresses the MyD88-dependent signalling network by targeting the ubiquitin system. Biochem. J..

[52] Youm Y.-H., Nguyen K.Y., Grant R.W., Goldberg E.L., Bodogai M., Kim D., D’agostino D., Planavsky N., Lupfer C., Kanneganti T.D. (2015). The ketone metabolite β-hydroxybutyrate blocks NLRP3 inflammasome–mediated inflammatory disease. Nat. Med..

[53] Boxer M.B., Shen M., Auld D.S., Wells J.A., Thomas C.J. (2010). A small molecule inhibitor of Caspase 1. Probe Reports from the NIH Molecular Libraries Program.

[54] Kuwar R., Rolfe A., Di L., Xu H., He L., Jiang Y., Zhang S., Sun D. (2019). A novel small molecular NLRP3 inflammasome inhibitor alleviates neuroinflammatory response following traumatic brain injury. J. Neuroinflamm..

[55] Saadane A., Masters S., DiDonato J., Li J., Berger M. (2007). Parthenolide inhibits IκB kinase, NF-κB activation, and inflammatory response in cystic fibrosis cells and mice. Am. J. Respir. Cell Mol. Biol..

[56] Sandall C.F., Ziehr B.K., MacDonald J.A. (2020). ATP-Binding and Hydrolysis in Inflammasome Activation. Molecules.

[57] Alehashemi S., Goldbach-Mansky R. (2020). Human Autoinflammatory Diseases Mediated by NLRP3-, Pyrin-, NLRP1-, and NLRC4-Inflammasome Dysregulation Updates on Diagnosis, Treatment, and the Respective Roles of IL-1 and IL-18. Front. Immunol..

[58] Walle L.V., Stowe I.B., Šácha P., Lee B.L., Demon D., Fossoul A., Van Hauwermeiren F., Saavedra P.H., Šimon P., Šubrt V. (2019). MCC950/CRID3 potently targets the NACHT domain of wild-type NLRP3 but not disease-associated mutants for inflammasome inhibition. PLoS Biol..

[59] Yang G., Lee H.E., Moon S.J., Ko K.M., Koh J.H., Seok J.K., Min J.K., Heo T.H., Kang H.C., Cho Y.Y. (2020). Direct Binding to NLRP3 Pyrin Domain as a Novel Strategy to Prevent NLRP3-Driven Inflammation and Gouty Arthritis. Arthritis Rheumatol..

[60] Maharana J., Vats A., Gautam S., Nayak B.P., Kumar S., Sendha J., De S. (2017). POP1 might be recruiting its type-Ia interface for NLRP3-mediated PYD-PYD interaction: Insights from MD simulation. J. Mol. Recognit..

[61] Tapia-Abellán A., Angosto-Bazarra D., Martínez-Banaclocha H., de Torre-Minguela C., Cerón-Carrasco J.P., Pérez-Sánchez H., Arostegui J.I., Pelegrin P. (2019). MCC950 closes the active conformation of NLRP3 to an inactive state. Nat. Chem. Biol..

[62] Sebastian-Valverde M., Wu H., Al Rahim M., Sanchez R., Kumar K., De Vita R.J., Pasinetti G.M. (2021). Discovery and characterization of small molecule inhibitors of NLRP3 and NLRC4 inflammasomes. J. Biol. Chem..

[63] Abdullaha M., Ali M., Kour D., Kumar A., Bharate S.B. (2020). Discovery of benzo [cd] indol-2-one and benzylidene-thiazolidine-2, 4-dione as new classes of NLRP3 inflammasome inhibitors via ER-β structure based virtual screening. Bioorg. Chem..

[64] Kinra M., Joseph A., Nampoothiri M., Arora D., Mudgal J. (2021). Inhibition of NLRP3-inflammasome mediated IL-1β release by phenylpropanoic acid derivatives: In-silico and in-vitro approach. Eur. J. Pharm. Sci..

[65] Yammine A., Gao J., Kwan A.H. (2019). Tryptophan fluorescence quenching assays for measuring protein-ligand binding affinities: Principles and a practical guide. Bio-Protoc..

[66] Irwin J.J., Shoichet B.K. (2005). *ZINC*—A free database of commercially available compounds for virtual screening. J. Chem. Inf. Model..

[67] Berman H.M., Battistuz T., Bhat T.N., Bluhm W.F., Bourne P.E., Burkhardt K., Feng Z., Gilliland G.L., Iype L., Jain S. (2002). The protein data bank. Acta Crystallogr. Sect. D Biol. Crystallogr..

[68] Sanner M.F. (1999). Python: A programming language for software integration and development. J. Mol. Graph. Model..

[69] Trott O., Olson A.J. (2010). AutoDock Vina: Improving the speed and accuracy of docking with a new scoring function, efficient optimization, and multithreading. J. Comput. Chem..

[70] Pettersen E.F., Goddard T.D., Huang C.C., Couch G.S., Greenblatt D.M., Meng E.C., Ferrin T.E. (2004). UCSF Chimera—A visualization system for exploratory research and analysis. J. Comput. Chem..

[71] DeLano W.L. (2002). The PyMOL Molecular Graphics System. https://pymol.org/2/.

[72] De Nardo C.M., Latz E. (2013). The Inflammasome: Methods and Protocols.

