# Supplementary material for: Identification of NLRP3PYD Homo-Oligomerization Inhibitors with Anti-Inflammatory Activity
Source: Int J Mol Sci. 2022 Jan 31;23(3):1651. doi: 10.3390/ijms23031651 (PMC8835912; doi:10.3390/ijms23031651)
Supplement: Supplementary file 1 [file ijms-23-01651-s001.zip › ijms-1532420-supplementary.pptx]

## Slide 1
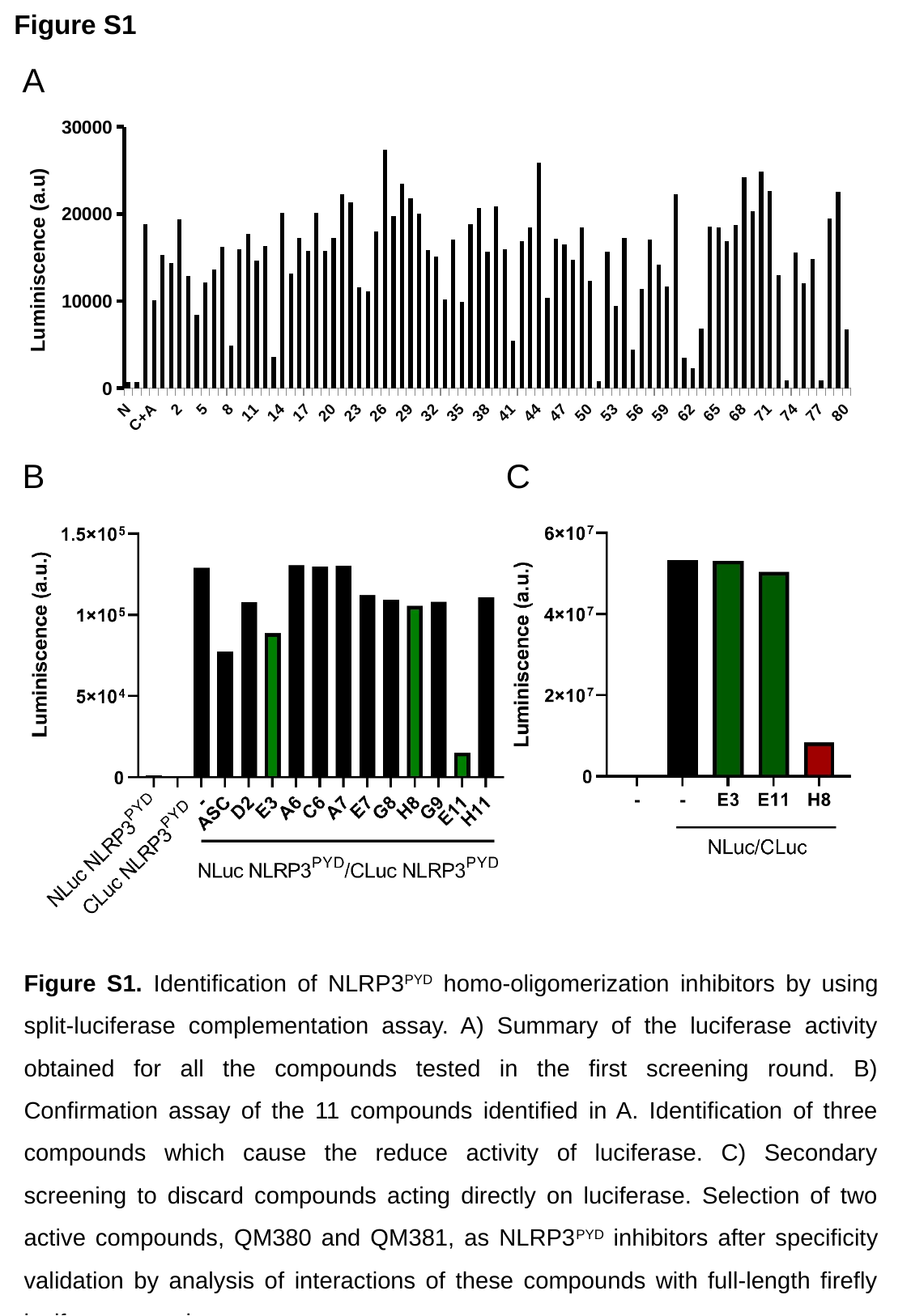

Figure S1
A
### Chart
| Category | |
|---|---|
| N | 622.5 |
| C | 623.75 |
| C+N | 18799.25 |
| C+ASC+N | 10059.0 |
| C+BSA+N | 15261.999999999998 |
| 1 | 14313.0 |
| 2 | 19318.0 |
| 3 | 12836.000000000002 |
| 4 | 8388.0 |
| 5 | 12067.0 |
| 6 | 13551.0 |
| 7 | 16199.0 |
| 8 | 4845.0 |
| 9 | 15873.0 |
| 10 | 17668.0 |
| 11 | 14602.0 |
| 12 | 16247.0 |
| 13 | 3504.0 |
| 14 | 20123.0 |
| 15 | 13128.0 |
| 16 | 17211.0 |
| 17 | 15705.0 |
| 18 | 20090.0 |
| 19 | 15691.000000000002 |
| 20 | 17195.0 |
| 21 | 22230.0 |
| 22 | 21268.0 |
| 23 | 11541.000000000002 |
| 24 | 11105.0 |
| 25 | 17931.0 |
| 26 | 27346.0 |
| 27 | 19679.0 |
| 28 | 23444.0 |
| 29 | 21730.0 |
| 30 | 19987.0 |
| 31 | 15837.0 |
| 32 | 15074.0 |
| 33 | 10170.0 |
| 34 | 17043.0 |
| 35 | 9853.0 |
| 36 | 18807.0 |
| 37 | 20609.0 |
| 38 | 15579.0 |
| 39 | 20864.0 |
| 40 | 15878.999999999998 |
| 41 | 5435.0 |
| 42 | 16868.0 |
| 43 | 18424.0 |
| 44 | 25874.0 |
| 45 | 10291.000000000002 |
| 46 | 17146.0 |
| 47 | 16486.0 |
| 48 | 14688.0 |
| 49 | 18422.999999999996 |
| 50 | 12271.000000000002 |
| 51 | 747.0 |
| 52 | 15608.0 |
| 53 | 9363.0 |
| 54 | 17221.0 |
| 55 | 4334.0 |
| 56 | 11350.0 |
| 57 | 17039.0 |
| 58 | 14102.0 |
| 59 | 11613.0 |
| 60 | 22249.0 |
| 61 | 3444.0 |
| 62 | 2216.0 |
| 63 | 6796.0 |
| 64 | 18489.999999999996 |
| 65 | 18427.0 |
| 66 | 16856.0 |
| 67 | 18694.0 |
| 68 | 24121.0 |
| 69 | 20231.0 |
| 70 | 24838.0 |
| 71 | 22606.000000000004 |
| 72 | 12967.0 |
| 73 | 852.0 |
| 74 | 15519.0 |
| 75 | 12008.0 |
| 76 | 14776.0 |
| 77 | 837.0 |
| 78 | 19402.0 |
| 79 | 22518.999999999996 |
| 80 | 6660.0 |Luminiscence (a.u)
B
C
Figure S1. Identification of NLRP3PYD homo-oligomerization inhibitors by using split-luciferase complementation assay. A) Summary of the luciferase activity obtained for all the compounds tested in the first screening round. B) Confirmation assay of the 11 compounds identified in A. Identification of three compounds which cause the reduce activity of luciferase. C) Secondary screening to discard compounds acting directly on luciferase. Selection of two active compounds, QM380 and QM381, as NLRP3PYD inhibitors after specificity validation by analysis of interactions of these compounds with full-length firefly luciferase protein.

## Slide 2
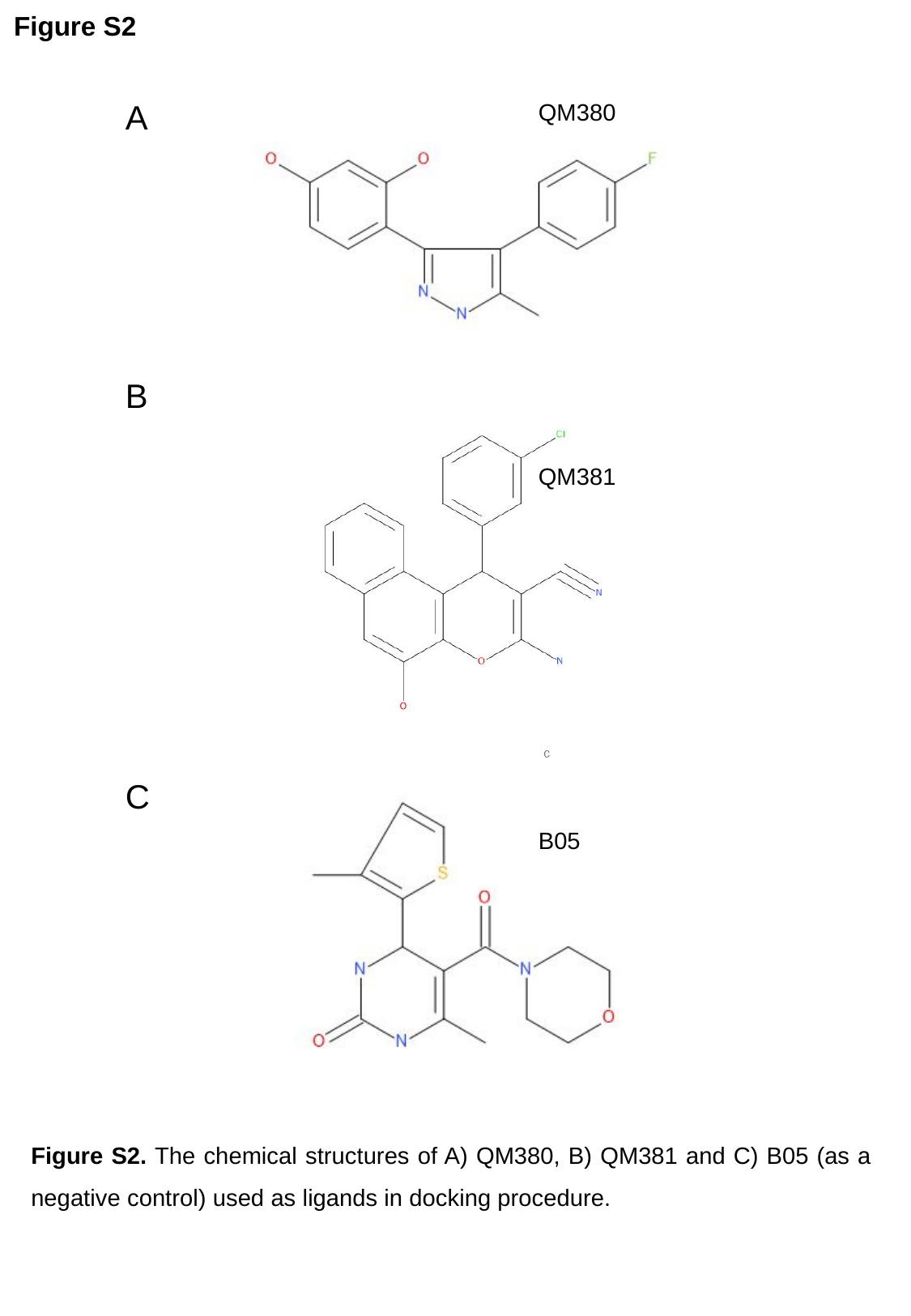

Figure S2
A
QM380
B
QM381
C
B05
Figure S2. The chemical structures of A) QM380, B) QM381 and C) B05 (as a negative control) used as ligands in docking procedure.

## Slide 3
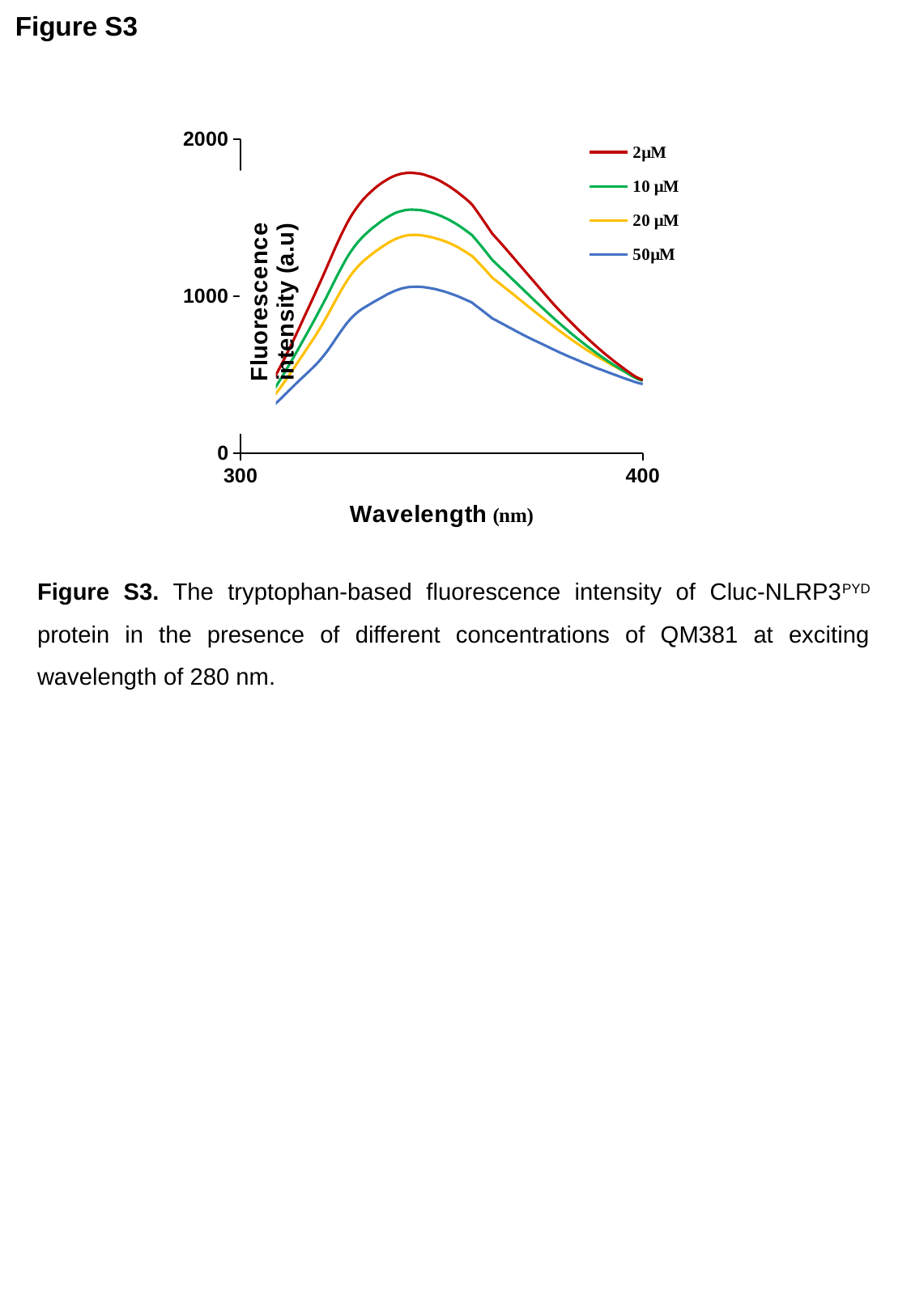

Figure S3
### Chart
| Category | 2μM | 10 μM | 20 μM | 50μM |
|---|---|---|---|---|Figure S3. The tryptophan-based fluorescence intensity of Cluc-NLRP3PYD protein in the presence of different concentrations of QM381 at exciting wavelength of 280 nm.

## Slide 4
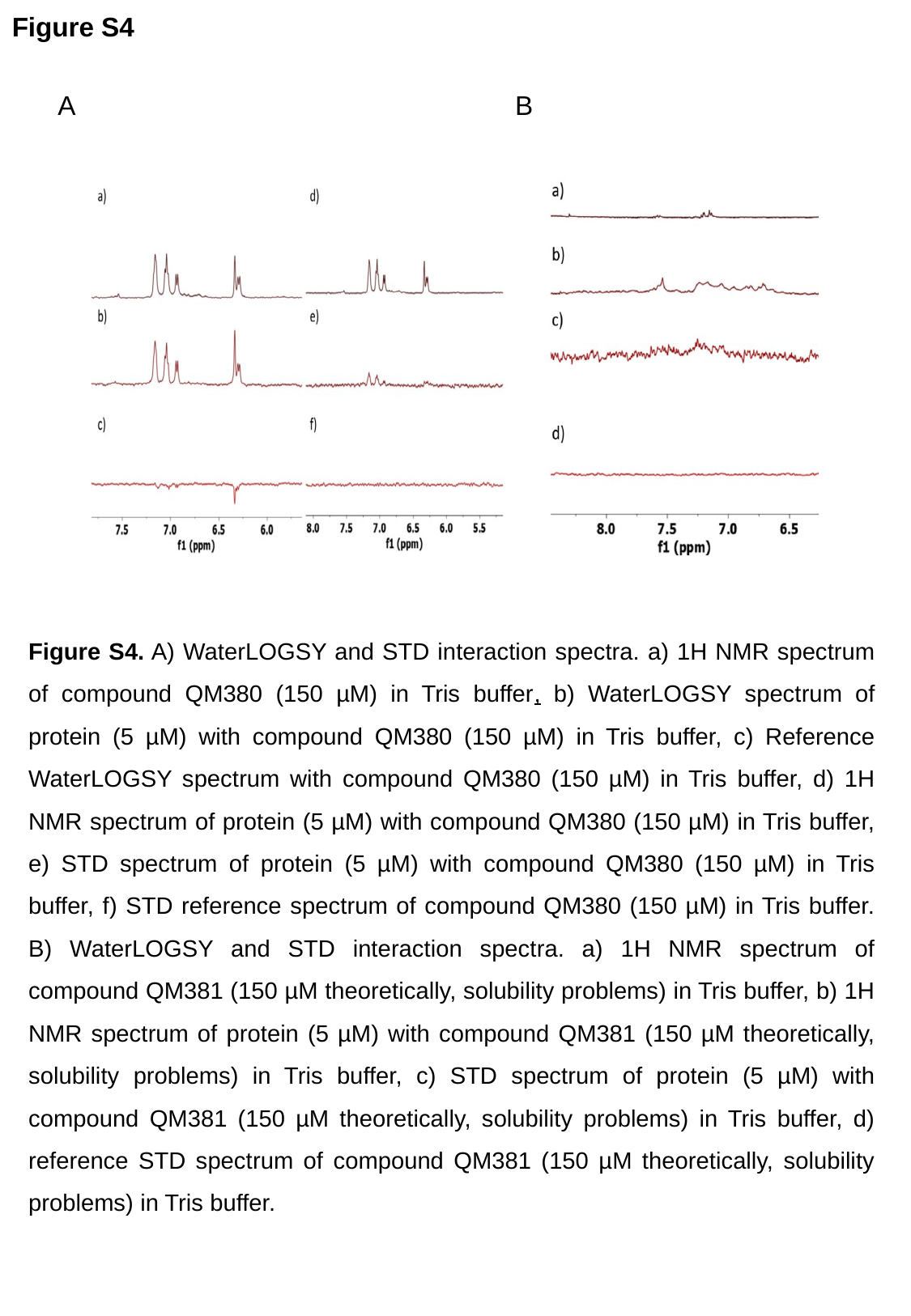

Figure S4
A
B
Figure S4. A) WaterLOGSY and STD interaction spectra. a) 1H NMR spectrum of compound QM380 (150 µM) in Tris buffer, b) WaterLOGSY spectrum of protein (5 µM) with compound QM380 (150 µM) in Tris buffer, c) Reference WaterLOGSY spectrum with compound QM380 (150 µM) in Tris buffer, d) 1H NMR spectrum of protein (5 µM) with compound QM380 (150 µM) in Tris buffer, e) STD spectrum of protein (5 µM) with compound QM380 (150 µM) in Tris buffer, f) STD reference spectrum of compound QM380 (150 µM) in Tris buffer. B) WaterLOGSY and STD interaction spectra. a) 1H NMR spectrum of compound QM381 (150 µM theoretically, solubility problems) in Tris buffer, b) 1H NMR spectrum of protein (5 µM) with compound QM381 (150 µM theoretically, solubility problems) in Tris buffer, c) STD spectrum of protein (5 µM) with compound QM381 (150 µM theoretically, solubility problems) in Tris buffer, d) reference STD spectrum of compound QM381 (150 µM theoretically, solubility problems) in Tris buffer.

## Slide 5
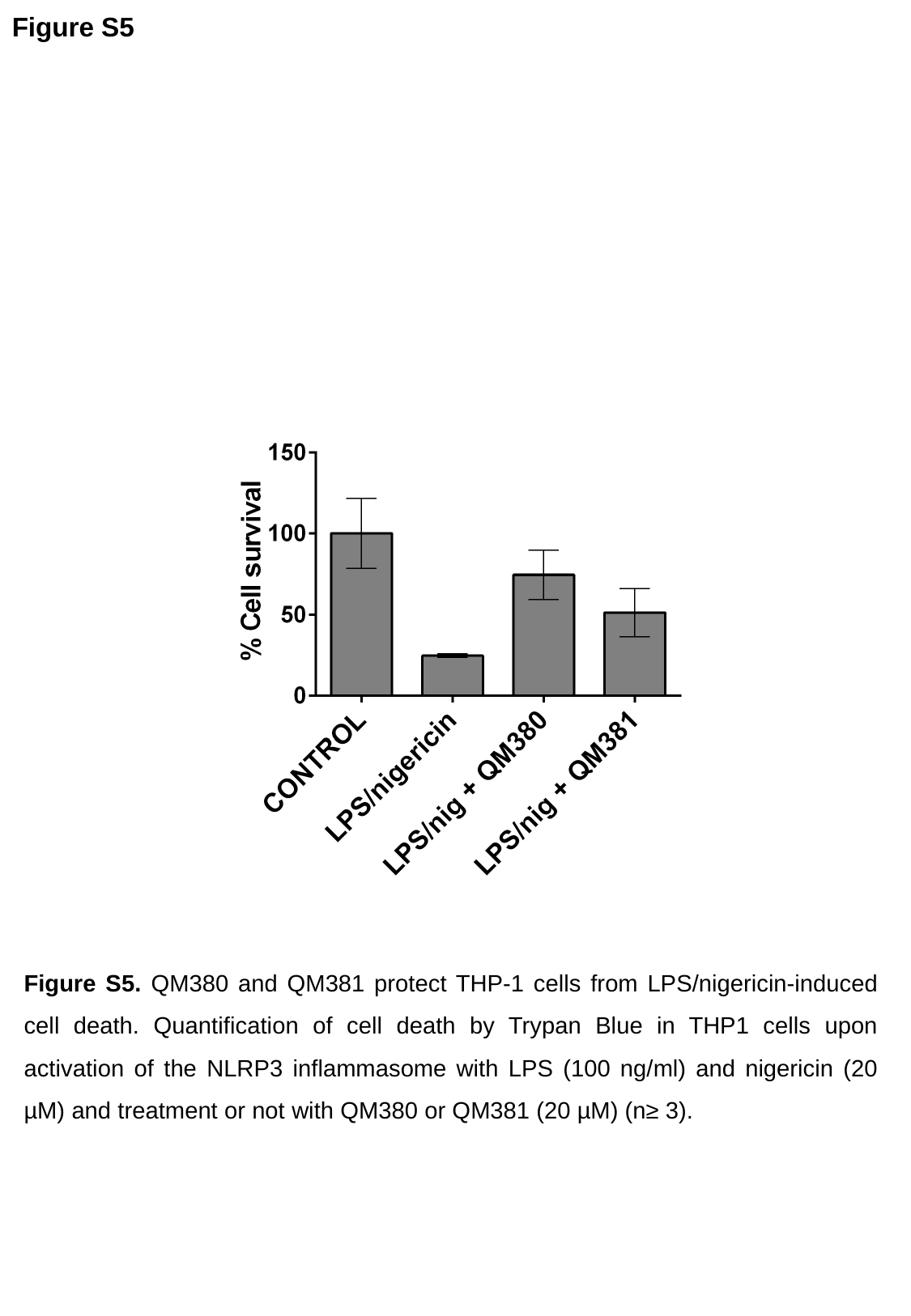

Figure S5
Figure S5. QM380 and QM381 protect THP-1 cells from LPS/nigericin-induced cell death. Quantification of cell death by Trypan Blue in THP1 cells upon activation of the NLRP3 inflammasome with LPS (100 ng/ml) and nigericin (20 µM) and treatment or not with QM380 or QM381 (20 µM) (n≥ 3).
